# Correlates of Successful Enrollment of Same-Sex Male Couples Into a Web-Based HIV Prevention Research Study: Cross-Sectional Study

**DOI:** 10.2196/15078

**Published:** 2020-01-09

**Authors:** Rob Stephenson, Tanaka MD Chavanduka, Stephen Sullivan, Jason W Mitchell

**Affiliations:** 1 The Center for Sexuality and Health Disparities School of Nursing University of Michigan Ann Arbor, MI United States; 2 Department of Systems, Population and Leadership School of Nursing University of Michigan Ann Arbor, MI United States; 3 Office of Public Health Studies Myron B Thompson School of Social Work University of Hawai’i at Manoa Honolulu, HI United States

**Keywords:** online research, dyadic, couples, recruitment

## Abstract

**Background:**

The recognition of the role of primary partners in HIV transmission has led to a growth in dyadic-focused HIV prevention efforts. The increasing focus on male couples in HIV research has been paralleled by an increase in the development of interventions aimed at reducing HIV risk behaviors among male couples. The ability to accurately assess the efficacy of these interventions rests on the ability to successfully enroll couples into HIV prevention research.

**Objective:**

This study aimed to explore factors associated with successful dyadic engagement in Web-based HIV prevention research using recruitment and enrollment data from a large sample of same-sex male couples recruited online from the United States.

**Methods:**

Data came from a large convenience sample of same-sex male couples in the United States, who were recruited through social media venues for a Web-based, mixed method HIV prevention research study. The analysis examined the demographic factors associated with successful dyadic engagement in research, measured as both members of the dyad meeting eligibility criteria, consenting for the study, and completing all study processes.

**Results:**

Advertisements generated 221,258 impressions, resulting in 4589 clicks. Of the 4589 clicks, 3826 individuals were assessed for eligibility, of which 1076 individuals (538/1913, 28.12% couples) met eligibility criteria and were included in the study. Of the remaining 2740 ineligible participants, 1293/3826 (33.80%) were unlinked because their partner did not screen for eligibility, 48/2740 (1.75%) had incomplete partner data because at least one partner did not finish the survey, 22/2740 (0.80%) were ineligible because of 1 partner not meeting the eligibility criteria. Furthermore, 492/3826 (12.86%) individuals were fraudulent. The likelihood of being in a matched couple varied significantly by race and ethnicity, region, and relationship type. Men from the Midwest were less likely to have a partner who did not complete the survey. Men with college education and those who labeled their relationships as husband or other (vs boyfriend) were more likely to have a partner who did not complete the survey.

**Conclusions:**

The processes used allowed couples to independently progress through the stages necessary to enroll in the research study, while limiting opportunities for coercion, and resulted in a large sample with relative diversity in demographic characteristics. The results underscore the need for additional considerations when recruiting and enrolling, relative to improving the methods associated with these research processes.

## Introduction

### Background

There is now substantial evidence for the role of male dyads in the US HIV epidemic, with primary partners identified as the source of approximately one-third [1] to two-thirds [2] of new HIV infections. Given these estimates, a significant paradigm shift in HIV prevention is needed. Programmatic efforts have traditionally focused on men who have sex with men (MSM), in particular, gay-identifying men as individuals rather than dyads, with a focus on casual sex as a risk for HIV acquisition. As a result of this individualistic approach, HIV prevention efforts have largely ignored the risk of HIV transmission that occurs within primary partnerships. Within the context of same-sex male couples’ relationships, various research findings have illustrated high rates of sexual risk behavior for HIV (with primary and casual partners), low rates of disclosure of potentially risky episodes with casual partners to primary partners, and reduced frequency of HIV testing [3-9]. Historically, HIV prevention efforts have focused on reducing the number of casual sex partners [10], indirectly messaging a false sense of protection associated with primary partners [11,12].

There have been recent attempts to address this disproportionate focus on individualistic approaches to HIV prevention by focusing on the dyads and their relationship. Couples HIV testing and counseling (CHTC), originally developed for heterosexual couples in sub-Saharan Africa [13], has been adapted for same-sex male couples [14], as a Centers for Disease Control and Prevention Public Health Strategy [15]. There are several examples of dyadic interventions that aim to address HIV risk among same-sex male couples. Connect with Pride was an intervention for methamphetamine-using, black/African American male dyads that involved 7 in-person sessions to address issues, such as communication, joint problem solving, and condom negotiation [16]. 2GETHER was an intervention for young male couples aged 18 to 29 years, which involved 4 interactive weekly sessions focusing on enhancing communication skills, coping with relationship stress, applying problem-solving techniques to relationship issues, and formulating an agreement to reduce their risk for HIV [17]. Posttest decreases in sexual risk behaviors, increases in skills related to HIV prevention, and improvements in relationship investment were observed. We Prevent is a novel intervention for 15- to 19-year-old male dyads, which is currently being piloted in the United States [18], involving telehealth-delivered sessions to increase relationship communication skills around HIV prevention. The Male Couples Agreement Project is an electronic health tool kit intervention for male couples with a foundation in relationship science, including sexual agreements, sexual health education, and HIV prevention [19,20], and Stronger Together focuses on improving engagement in HIV care and antiretroviral treatment adherence among serodiscordant male couples by combining in-person CHTC with dyadic adherence counseling [21]. Although these interventions have the potential to improve relational dynamics and safeguard male couples against HIV and other sexually transmitted infections, the ability to successfully test the efficacy of these interventions rests on the ability to successfully enroll male couples into HIV prevention research. Representation of diverse samples of same-sex male couples—in terms of age, race, ethnicity, and relationship length—in dyadic HIV prevention interventions is critical for measuring the success of these projects and moving forward toward improving them.

Few studies have addressed the challenges encountered in recruiting same-sex male couples into research. To enroll a couple into research requires both members of the couple to successfully navigate parallel processes: both must screen for eligibility, provide consent, and complete some other data collection activity (eg, study survey) to enroll into a research project. These processes must be conducted separately to address and prevent coercion among partners to participate in research projects, especially when there are financial incentives for participation. Successful participation often requires the couple to share information (ie, partner A must inform partner B that they have completed their consent form) or to coordinate (ie, they must jointly schedule a study visit). At a minimum, both members of the couple must agree to participate in the research study, knowing that their partner will also be participating. These processes may result in studies obtaining a potentially selective group of couples with more functional communication styles or couples with reduced levels of conflict. In their study of 260 partnered gay/bisexual men recruited in New York City, Starks et al [22] found those who did not refer their partners were older, wealthier, and in longer relationships, whereas participants who successfully recruited their partners were significantly more satisfied in their relationship. This selectivity is important to consider in light of evidence illustrating associations between poor relationship characteristics and HIV prevention outcomes [3,5,23-27].

### Objective

Missing from the literature is an understanding of the factors associated with successful enrollment of same-sex male couples in Web-based HIV prevention research studies. Although limited research has identified relationship factors associated with referring partners into Web-based HIV research, other factors associated with successful dyadic engagement in other parts of the research process (ie, eligibility and consent) have yet to be investigated. In general, Web-based studies may be associated with higher degrees of selectivity bias, with recent evidence suggesting that white MSM are more likely to join a Web-based study than black and Hispanic MSM [28]. The identification of such selectivity biases is equally important for Web-based research with couples. If Web-based HIV prevention research is to be successful in identifying unique risk factors or prevention opportunities for same-sex male couples, then research must be able to successfully enroll diverse samples of couples. This study used recruitment and enrollment data from a large sample of same-sex male couples, recruited online from the United States, to explore factors associated with successful dyadic enrollment in Web-based HIV prevention research. This new information has the potential to shape recruitment and research designs for enrollment of dyadic HIV research.

## Methods

### Recruitment and Inclusion Criteria

Project Couples Health and Attitudes toward Preexposure Prophylaxis (CHAPS) is a mixed method Web-based study examining attitudes toward pre-exposure prophylaxis (PrEP) use and patterns of PrEP use among concordant seronegative and serodiscordant same-sex male couples in the United States. Participants were recruited through targeted Web-based advertisements and postings on commonly used social media websites and dating websites and mobile apps. Social media websites used for recruitment were Facebook and Instagram. Dating websites and mobile apps used for recruitment were Scruff and Grindr. Advertisements included images of a diverse (in age, race, and ethnicity) range of same-sex male couples, with text that promoted a study on the health of same-sex male couples (ie, Are you and your man on the same page about HIV prevention? We want to know, take our survey!). The advertisements did not mention PrEP to avoid recruiting a sample biased toward those with particular interests in or attitudes about PrEP. The advertisements included a link that led interested individuals to a landing page with detailed information about the study and a Web-based eligibility screener.

First, individual-level eligibility was established for both partners of the couple, and this had to have been met by both for enrollment. Individual eligibility self-reporting as (1) a cisgender male (assigned male at birth and currently identifies as male), (2) being in a relationship with another cisgender male for 3 or more months, (3) having an HIV seronegative or unknown status or known HIV seropositive status, and (4) having had condomless anal sex with their primary relationship partner within the last 3 months. Once eligible, an individual would then proceed to the consent webpage outlining the content and process of the study. Once consent was provided, the individual (partner A) would then be directed to the partner referral system, which entailed providing contact information (email and telephone number) and a name or nickname for his partner (partner B). Partner B would then receive an email informing him that his partner (partner A) had signed up for the study and had provided his contact information, along with a link to a landing page to access the same screener and consent process.

The link provided to partner B was connected to partner A’s metadata, such that they both were assigned the same random study ID number as a hidden data field (as a couple). Once partner B had completed the same eligibility screener and consent process, partner B was then asked to provide contact information for his partner (partner A), to enable crossmatching of partner contact details.

Couple serostatus was also considered. Given the focus on PrEP, only concordant seronegative and serodiscordant couples were eligible. Once both A and B had completed the screener, their responses to the question on serostatus were compared. Couples who reported concordant seropositive status were deemed ineligible for the study.

Following successful completion of the eligibility and consent process by both partners A and B, as well as identification of concordant seronegative or serodiscordant HIV status, individual emails were sent to each partner of the couple, asking them to independently and individually complete a Web-based survey via a link. The survey Web link contained the same random study ID number they were assigned during eligibility screener to help link partners A and B’s completed survey responses. Each partner was compensated US $50 for his time to complete the survey; compensation was not dependent on both partners completing the survey. The study protocol was approved by the University of Michigan Institutional Review Board (HUM00125711).

### Matching and Verification of Participants

Upon completion of their individual surveys, couples’ responses were compared with verify that they were real couples. First, individual surveys were linked as couples via the identifier included in the survey link. Couple status was verified using the relationship and contact information provided by each individual. A verified couple had to match on at least 4 of the following 6 criteria (identified through questions asked in the eligibility screener): (1) partner’s age (± 1 year), (2) partner’s birthday month, (3) relationship length, (4) anal sex without a condom within the last 3 months, (5) initials of partner’s first and last name, and (6) last 4 digits of partner’s cell phone number. Matching of couple data was manually reviewed and checked for matching: each couple was assigned a score from 1 to 6, which represented the number of criteria on which they matched in their surveys.

#### Detecting Fraudulent Activity

All participant data were also manually reviewed and checked for mismatch, duplication, and fraud. Inconsistent information, such as name, internet protocol address, zip code, email, or phone number, was flagged for further inquiry. Participants were contacted directly by study staff for confirmation of their identity and relationship status. Individuals were classified as fraudulent if their identity could not be verified.

#### Match Status Categories

On the basis of the results of verification and matching, participants were categorized into 4 groups: eligible couple, incomplete, ineligible, and unlinked. *Eligible couples* comprised couples in which both relationship partners were eligible, consented, passed verification, and completed the study survey. *Incompletes* included couples in which 1 or both partners did not finish the study survey. *Ineligibles* were couples in which 1 partner met individual-level eligibility criteria and consented, whereas the other partner either did not meet this eligibility criteria or did not consent. *Unlinked* was defined as cases in which only 1 partner completed the enrollment process (eligible, consented, and completed the study survey), whereas the other did not enter the screening and enrollment process.

### Study Survey

The survey was distributed via Qualtrics (Qualtrics International Inc) through an anonymous link embedded with a unique identifier that linked couples, and it took partners, on average, 35 min to complete. The survey contained a variety of measures geared toward understanding the dyadic patterns relative to PrEP. The aim of this study was to examine the factors associated with achieving a successfully matched and verified couple recruited online. The analysis models a 4-category outcome variable, representing the 4 possibilities encountered from enrollment: eligible couples (the reference category), ineligible, incomplete, and unlinked. Data analysis comprised individual-level data in which every line of data is an individual, to facilitate the inclusion of participants for whom no partner data were received (eg, unlinked). A multinomial model is fit for the 4-category matching status outcome. Key covariates included the sociodemographic characteristics of individuals: education, employment, housing status, race and ethnicity, age, relationship length, and relationship type. Relationship type was categorized to compare more informal relationship types (boyfriend or other) with more formal relationship types (husband or partner). The analysis was conducted in STATA v.15 [29].

## Results

CHAPS advertisements generated 221,258 impressions (number of times it was shown on a social medial page), resulting in 4589 clicks (number of times the advertisement was clicked on: these may not be unique to individuals). Of the 4589 clicks, 3826 individuals were assessed for eligibility, of which 1076 individuals (538/1913, 28.12% couples) were matched eligible and included in the study. Of the remaining 2740 participants, 1293/3826 (33.80%) were unlinked because of their partner not enrolling into the screening, 48/2740 (1.75%) had incomplete partner data because at least 1 partner did not finish the survey, 22/2740 (0.80%) were ineligible because of 1 partner not meeting the eligibility criteria. Furthermore, 492/3826 (12.86%) individuals were fraudulent, and 885/3826 (23.13%) started the screening but did provide any responses; therefore, they were deemed invalid. Fraudulent and invalid participants were removed from the sample, resulting in a sample of 2449 individuals, which includes 538 eligible and verified couples. Those who had missing data (n=911) from key covariates, including region, education, housing, and relationship length, were dropped from dataset, resulting in a final analysis sample of 1538 individuals.

Characteristics of the analysis sample (N=1538) are described in Table 1.

The sample was largely white (1140/1538, 74.12%) and between the ages of 25 and 34 years (875/1538, 56.89%). A majority of participants were from the South (495/1538, 32.18%) and the Midwest (457/1538, 29.71%) regions. A majority of individuals were college graduates (521/1538, 33.86%) or had graduate degrees (392/1538, 25.48%), worked full time (1215/1538, 79.00%), and lived in their own housing (1237/1538, 80.42%). Finally, 35.89% (552/1538) of the sample identified as boyfriends and 34.39% (529/1538) identified as husbands, with the largest portion of relationship lengths being between 1 and 3 years (494/1538, 32.12%) and more than 5 years (524/1538, 34.07%).

White-Hispanic participants were significantly more likely to have a partner who was ineligible for the study (relative risk ratio [RRR]=4.94; 95% CI 1.15-21.26) or to be in the incomplete partner status (RRR=2.55; 95% CI 1.02-6.42) compared with being in the eligible couple category. Those who reported being from the Midwest (RRR=0.25; 95% CI 0.08-0.72) were less likely to be in the incomplete category than have an eligible partner. Men with a college education or above (RRR=2.82; 95% CI 1.02-7.80) and having a husband/partner for a relationship type (RRR=2.35; 95% CI 1.06-5.25) were more likely to be in the incomplete category than have an eligible partner. There were no significant associations with the remaining covariates across the match status outcomes.

Results of the multinomial model are shown in Table 2.

**Table 1:** Descriptive statistics for participants and demographic variables (N=1538).

Characteristics		Eligible individuals^a^ (n=1076), n (%)	Ineligible partners^b^ (n=12), n (%)	Incomplete partner^c^ (n=35), n (%)	Unlinked participant^d^ (N=415), n (%)
**Race/ethnicity**					
	Non-Hispanic white	804 (74.72)	7 (58)	25 (71)	303 (73.0)
	Other^e^	197 (18.31)	2 (17)	3 (9)	73 (17.6)
	White Hispanic	75 (6.97)	3 (25)	7 (20)	39 (9.4)
**Age (years)**					
	18-24	160 (14.87)	5 (42)	7 (20)	83 (20.0)
	25-34	630 (58.55)	4 (33)	19 (54)	222 (53.5)
	35-44	215 (19.98)	2 (17)	7 (20)	91 (21.9)
	45+	71 (6.60)	1 (8)	2 (6)	19 (4.6)
**Region**					
	Northeast	186 (17.29)	1 (8)	12 (34)	70 (16.9)
	South	333 (30.95)	4 (33)	10 (29)	148 (35.7)
	West	220 (20.45)	2 (17)	8 (23)	88 (21.2)
	Midwest	337 (31.32)	5 (42)	5 (14)	109 (26.3)
**Education**					
	Up to high school/some college	321 (29.83)	6 (50)	5 (14)	152 (36.6)
	College/some graduate school	755 (70.17)	6 (50)	30 (86)	263 (63.4)
**Employment**					
	Work full time	857 (79.65)	8 (67)	24 (69)	326 (78.6)
	Work part time/retired	219 (20.35)	4 (33)	11 (31)	89 (21.5)
**Housing**					
	My own house or apartment	877 (81.51)	8 (67)	30 (86)	322 (77.6)
	Other^f^	199 (18.49)	4 (33)	5 (14)	93 (22.4)
**Relationship type**					
	Boyfriend/other^g^	645 (59.94)	8 (67)	15 (43)	244 (58.8)
	Husband/partner	431 (40.06)	4 (33)	20 (57)	171 (41.2)
**Relationship length**					
	More than 3 months but less than 1 year	132 (12.27)	3 (25)	4 (11)	60 (14.5)
	More than 1 year but less than 3 years	347 (32.25)	5 (42)	7 (20)	134 (32.3)
	More than 3 years but less than 5 years	228 (21.19)	1 (8)	12 (34)	81 (19.5)
	More than 5 years	369 (34.29)	3 (25)	12 (34)	140 (33.7)

^a^Eligible couples: couples in which both relationship partners were eligible, consented, passed verification, and completed the study survey.

^b^Ineligibles: couples in which 1 partner met individual-level eligibility criteria and consented, whereas the other partner either did not meet the eligibility criteria or did not consent.

^c^Incompletes: included couples in which 1 or both partners did not finish the study survey.

^d^Unlinked was defined as cases in which only 1 partner completed the enrollment process (eligible, consented, and completed the study survey), whereas the other did not enter the screening and enrollment process.

^e^Includes 72 black and African American, 72 mixed, 64 Hispanic and Latino, 47 Asian, 7 Native American and Alaskan Native, 5 Middle Eastern, 5 Native Hawaiian and Other, Pacific Islander, 1 Caribbean, 1 Southern European, and 1 Indian.

^f^Includes college dorm, employee housing, and sharing with significant other.

^g^Includes friends with benefits, mates, best friend, bae, and better half.

**Table 2:** Multinomial logistic regression results for couple match status (N=1538).

Characteristics		Ineligible partners vs eligible couples, RRR^a^ (95% CI)	Incomplete partner vs eligible couples, RRR (95% CI)	Unlinked participant vs eligible couples, RRR (95% CI)
**Race**				
	Non-Hispanic white	Ref^b^	Ref	Ref
	Other	1.08 (0.21-5.52)	0.51 (0.14-1.73)	0.93 (0.68-1.26)
	White Hispanic	4.94 (1.15-21.26)^c^	2.55 (1.02-6.42)^c^	1.27 (0.84-1.94)
**Age (years)**				
	18-24	Ref	Ref	Ref
	25-34	0.34 (0.07-1.58)	0.51 (0.19-1.40)	0.74 (0.53-1.04)
	35-44	0.54 (0.08-3.91)	0.58 (0.16-2.02)	0.88 (0.59-1.33)
	45+	0.85 (0.07-10.58)	0.51 (0.09-2.94)	0.55 (0.30-1.02)
**Region**				
	Northeast	Ref	Ref	Ref
	South	2.25 (0.24-21.10)	0.50 (0.21-1.21)	1.16 (0.83-1.64)
	West	1.39 (0.12-16.29)	0.55 (0.21-1.42)	0.99 (0.68-1.44)
	Midwest	2.87 (0.32-25.58)	0.25 (0.08-0.72)^c^	0.83 (0.59-1.19)
**Education**				
	Up to high school/some college	Ref	Ref	Ref
	College/some graduate school	0.71 (0.20-2.53)	2.82 (1.02-7.80)^c^	0.80 (0.62-1.03)
**Employment**				
	Work full time	Ref	Ref	Ref
	Work part time/retired	1.30 (0.36-4.71)	2.05 (0.94-4.45)	0.97 (0.72-1.30)
**Housing**				
	My own house or apartment	Ref	Ref	Ref
	Other	1.37 (0.35-5.28)	0.78 (0.28-2.16)	1.17 (0.87-1.58)
**Relationship type**				
	Boyfriend/other	Ref	Ref	Ref
	Husband/partner	1.07 (0.26-4.39)	2.35 (1.06-5.25)^c^	1.10 (0.85-1.44)
**Relationship length**				
	More than 3 months but less than 1 year	Ref	Ref	Ref
	More than 1 year but less than 3 years	0.75 (0.16-3.58)	0.64 (0.18-2.33)	0.90 (0.62-1.32)
	More than 3 years but less than 5 years	0.28 (0.02-3.18)	1.28 (0.37-4.47)	0.85 (0.56-1.30)
	More than 5 years	0.54 (0.06-4.56)	0.70 (0.18-2.75)	0.92 (0.60-1.41)

^a^RRR: relative risk ratio.

^b^Reference category.

^c^*P*<.05.

## Discussion

### Principal Findings

The results illustrate several important facets of the recruitment of same-sex male couples into Web-based HIV prevention research. First, advertising through social media to recruit couples generated 3826 individuals who completed the eligibility screener. Of these, only 538 couples (1076 individuals) were successfully engaged in research (eligible, consented, completed a survey, and were matched with their partner). Therefore, 72% of responses failed to generate successfully matched couples. This has significant resource implications given that the majority of social media recruitment involves paid advertising. Project CHAPS intentionally used images of same-sex male couples, which included representation of diverse ages, races, and ethnicities. These advertisements were based on those used previously to successfully enroll online samples of over 400 same-sex male couples in the United States [30]. However, further work is warranted to explore same-sex male couples’ perceptions and desired content for online recruitment advertising, to help facilitate the creation of advertisements with optimal appeal to help with enrollment into Web-based research studies.

Of importance, almost 34.00% (523/1538) of participants had partners who did not initiate the screening and enrollment process, whereas failure of having a partner not complete the survey only accounted for 2% (35/1538) of unmatched couples and a partner being ineligible accounted for less than 1% (12/1538) of the unmatched couples. Therefore, once both partners made it through the eligibility screener, there was a very high likelihood that they would become a successfully matched couple who would both complete the study survey. This suggests a need to strengthen partner referral methods early on in the study engagement process. Providing individuals with detailed information on the study that they can share with their partner, which clearly outlines the steps that their partner needs to take, is a fundamental step in increasing dyadic recruitment. Of course, this process must be careful not to cross over into coercion: systems need to be maintained, which allow both partners to independently screen and consent for studies.

The ability to identify and match couples was enhanced by the use of a series of fraud detection techniques, based on the standards recommended by Bauermeister et al [31]. An additional fraud technique was implemented, which is specific to the enrollment of dyads. Once surveys were completed, responses to 6 key questions regarding relationship and partner characteristics were compared: those who matched on fewer than 4 responses were deemed not to be a real couple. This form of couple verification has been recommended as a mechanism for reducing the degree of fraud in dyadic Web-based research [32]. However, further work is required to inform the content of couple verification surveys. Questions must represent a range of partner and relationship characteristics that partners may be expected to know, but these must also be sensitive enough to identify fraudulent couples.

Few factors were significantly associated with the successful engagement of male dyads, contrary to the work of Starks et al [22], which showed significant differences in partner referral into a research study be age, wealth, and relationship length. The likelihood of being an eligible couple versus having an ineligible partner, incomplete partner, or an unlinked partner did not vary by relationship length, suggesting our recruitment and enrollment methods were successful at engaging couples at range of relationship stages. Men who reported themselves as being in a more formal union (ie, husbands) were more likely to have a partner who did *not* complete the screening and enrollment process. This seems counter intuitive as it may be expected that those in more formal unions may have developed stronger, or at least more familiar, communication styles that may lend themselves to successful enrollment in HIV research. However, it is possible that engagement in research about relationships and/or HIV prevention may not be one of those shared interests and values among partners. Although further research is required to understand this result, ideally qualitative work that examines perceptions of enrolling in HIV prevention research from a range of couple types, it is possible that more formal and established couples do not see themselves as at risk for HIV and therefore do not see the research as being suitable for them. Previous research has identified that coupled MSM perceive lower levels of HIV risk [5], and this may shape how couples view their eligibility or desire to enroll in an HIV prevention study.

White-Hispanic men were more likely to have an ineligible partner or a partner who did not complete the screening process. This result may reflect the myriad of interpersonal and structural barriers that men of color experience in enrolling in research. The sample for this study is overwhelmingly non-Hispanic white, limiting the ability to understand whether the ability to enroll in a survey for male couples varies for racial and ethnic minority couples. It seems plausible that couples with African American men may also be more likely to face difficulties in enrolling as couples in HIV prevention research, but the very small number of African American men in this study precludes such analysis. The advertisements used for CHAPS included a diverse range of races and ethnicities; however, this still resulted in a predominantly non-Hispanic white sample. Further qualitative work—with diverse racial and ethnic samples of same-sex male couples—would be needed to fully understand the perceptions of Web-based dyadic HIV prevention research, as well as their needs and desires that would lead them to participate in future studies.

It is important to note that Project CHAPS was a cross-sectional survey and did not require the participants to take part in an intervention or to take follow-up surveys over a period of time. Although this study has identified factors associated with successful enrollment into a 1-time survey, it is likely that factors shaping the ability of couples to actively participate in intervention research may differ. This may be particularly true for interventions that require members of the dyad to take the intervention together (ie, couples’ counseling–focused interventions). There may also be differential follow-up over time among couples, with only 1 member of the couple completing follow-up surveys, leading to limitations to dyadic data analysis. Although this paper identifies processes for enrolling couples into surveys, further work is required to understand whether the process required for successful participation of couples in intervention-focused research differs.

### Conclusions

This study is not without limitations. Using cross-sectional data from a convenience sample precludes us from making causal inferences or generalizing our results to other same-sex male couples in the United States, who may or may not use social media platforms or geospatial mobile apps. The collection of personal identifying information may have prompted social desirability to inaccurately report data on their partners or relationship characteristics. Although participants were instructed to complete the survey separately from their partners, it is possible that couples answered questions together, potentially influencing each other’s responses and overestimating the degree to which couples truly matched their knowledge of each other and their relationship. The sample was overwhelmingly non-Hispanic white, limiting the ability to make inferences about specific strategies for enrolling racial and ethnic minority couples into HIV prevention research. Given the higher incidence of HIV among MSM of color, work is clearly needed to understand the barriers that racial-ethnic minority male couples may experience in enrolling and participating in HIV prevention research.

Despite these limitations, the results presented here provide important new information on the processes required to successfully enroll same-sex male couples into Web-based HIV prevention research. The steps used in CHAPS allowed couples to independently progress through the stages necessary to enroll in the research study while limiting opportunities for coercion and resulted in a large, diverse sample (>500 couples). The results underscore the need for additional considerations when recruiting and enrolling, relative to improving the methods associated with these research processes. Further research is needed and is beneficial to fully understand the perceptions of same-sex male couples toward Web-based research. This information is vital for the continued refinement of dyadic recruitment and engagement methods.

## Abbreviations

CHAPS: Couples Health and Attitudes toward Preexposure Prophylaxis
CHTC: Couples HIV testing and counseling
MSM: men who have sex with men
PrEP: pre-exposure prophylaxis
RRR: relative risk ratio
